# Duplication and Sub/Neofunctionalization of *Malvolio*, an Insect Homolog of *Nramp*, in the Subsocial Beetle *Nicrophorus vespilloides*

**DOI:** 10.1534/g3.117.300183

**Published:** 2017-08-22

**Authors:** Elijah C. Mehlferber, Kyle M. Benowitz, Eileen M. Roy-Zokan, Elizabeth C. McKinney, Christopher B. Cunningham, Allen J. Moore

**Affiliations:** * Department of Entomology, University of Georgia, Athens, Georgia 30602; † Department of Genetics, University of Georgia, Athens, Georgia 30602

**Keywords:** behavior, burying beetle, gene expression, parenting, phylogenetics

## Abstract

With growing numbers of sequenced genomes, increasing numbers of duplicate genes are being uncovered. Here we examine *Malvolio*, a gene in the natural resistance-associated macrophage protein (Nramp) family, that has been duplicated in the subsocial beetle, *Nicrophorus vespilloides*, which exhibits advanced parental behavior. There is only one copy of *Mvl* in honey bees and Drosophila, whereas in vertebrates there are two copies that are subfunctionalized. We first compared amino acid sequences for Drosophila, beetles, mice, and humans. We found a high level of conservation between the different species, although there was greater variation in the C-terminal regions. A phylogenetic analysis across multiple insect orders suggested that *Mvl* has undergone several independent duplications. To examine the potential for different functions where it has been duplicated, we quantified expression levels of *Mvl1* and *Mvl2* in eight tissues in *N. vespilloides*. We found that while *Mvl1* was expressed ubiquitously, albeit at varying levels, expression of *Mvl2* was limited to brain and midgut. Because *Mvl* has been implicated in behavior, we examined expression during different behavioral states that reflected differences in opportunity for social interactions and expression of parental care behaviors. We found differing expression patterns for the two copies, with *Mvl1* increasing in expression during resource preparation and feeding offspring, and *Mvl2* decreasing in these same states. Given these patterns of expression, along with the protein analysis, we suggest that *Mvl* in *N. vespilloides* has experienced sub/neofunctionalization following its duplication, and may be evolving differing and tissue-specific roles in behavior and physiology.

The process of gene duplication is one of the primary mechanisms hypothesized to play a role in the evolution of novel phenotypes (Ohno 1970; Ditmar and Liberles 2010; Innan and Kondrashov 2010; Wagner 2011). When duplicate genes are maintained, one copy often becomes free to mutate and acquire new functions, as it is no longer constrained by the selective pressure to perform its previous role (Ohno 1970; Maere and Van de Peer 2010). This process can take two nonexclusive paths: subfunctionalization or neofunctionalization (Nadeau and Sankoff 1997; Nowak *et al.* 1997; Wagner 1998; Force *et al.* 1999; Maere and Van de Peer 2010). In the former process, both genes lose a portion of their function, so that the two duplicated genes together recapitulate the function of the ancestral gene (Force *et al.* 1999; Maere and Van de Peer 2010). In the latter process, one duplicate evolves a novel function absent from the ancestral gene (Ohno 1970; Maere and Van de Peer 2010). Neofunctionalization may also arise following subfunctionalization (Maere and Van de Peer 2010). Given the proposed role that gene duplication has in the production of new phenotypes, it follows that more derived organisms with novel traits will provide good systems for investigating the divergence of duplicated genes. Genetic influences on behavior may require neofunctionalization of gene duplications to overcome constraints that would otherwise arise through pleiotropy. For example, G-protein coupled receptors are cell surface receptors where gene duplications have evolved to become diversified and specialized for different behaviors (Katz and Lillvis 2014). Gene duplication and neofunctionalization have been implicated in the evolution of insect behaviors as diverse as *vitellogenin*’s influences on ant queen and worker social behavior and tasks (Corona *et al.* 2013), olfactory receptors related to shifts to herbivory (Goldman-Huertas *et al.* 2015), and opsin genes related to color vision and foraging preferences (Feuda *et al.* 2016).

With the advent of improved bioinformatics and sequencing, we are acquiring information on genomes of nonmodel organisms at an accelerated pace, many of which are studied primarily for their derived novel traits and not for their genetic accessibility. Such genomes often reveal duplicated genes that may have previously been unknown. We recently sequenced, assembled, and annotated the genome of such an organism, the burying beetle *Nicrophorus vespilloides* (Cunningham *et al.* 2015b). Burying beetles (*Nicrophorus* spp.) are unusual among beetles for their parenting behavior. Burying beetles breed on vertebrate carrion, which they shape into a ball, prepare with antimicrobial secretions, and bury. The larvae then crawl onto the carcass and one or both parents care for the offspring. Parenting in burying beetles is more than provisioning food for developing offspring, as in many insects; it involves direct and extensive prolonged social interactions. Burying beetles not only prepare and maintain a carcass for food, they feed their offspring by regurgitating predigested carrion directly into their mouths. Parenting in this taxon is thus both complex and extensive, and strongly selected as it influences the fitness of offspring (Eggert *et al.* 1998; Lock *et al.* 2004). This behavior is highly derived, and therefore we predicted that sub/neofunctionalization could be important in the evolution of complex parenting. Therefore, we examined this genome for evidence of duplications.

A candidate duplicate gene we found in the genome of *N. vespilloides* is *Malvolio*, a transporter of divalent cations and homolog of the Nramp (natural resistance-associated macrophage proteins) family in vertebrates (Folwell *et al.* 2006). *Malvolio* has been ascribed a role in behavior as well as cation transport (Evans *et al.* 2001). Moreover, there is evidence that *Nramp1* and *Nramp2* have subfunctionalized in mammals and fish (Techau *et al.* 2007; Neves *et al.* 2011). In fish, for example, *Nramp1* has been lost but a duplication of *Nramp*2 and subsequent subfunctionalization recovers the primary roles of iron transport and defense against pathogens (Neves *et al.* 2011). These roles are achieved by one form with ubiquitous expression and the other copy with localized expression, primarily in the immune cells and neuronal cells (Searle *et al.* 1998; Evans *et al.* 2001; Techau *et al.* 2007; Neves *et al.* 2011). However, in the best-studied insects, honey bees and *Drosophila*, there is only one copy of *Malvolio*. This gene is ubiquitously expressed and believed to function in many of the same roles as the human homolog of *Nramp*2, but is also known to effect behavior (Rodrigues *et al.* 1995). *Mvl* influences the transition between nurse and forager roles in honey bees (Ben-Shahar *et al.* 2004; Søvik *et al.* 2015) and food choice in *Drosophila* (Orgad *et al.* 1998; Søvik *et al.* 2017). Given the role this gene plays in behavior, and its duplication in *N. vespilloides*, we hypothesize that it has undergone sub/neofunctionalization and plays a role in the unique social behavior of *N. vespilloides*.

To begin our investigation into whether the duplication of *Malvolio* in beetles facilitated sub/neofunctionalization in relation to its effects on behavior, we first examined *Malvolio* protein sequences across mammals and insects. We then built a gene phylogeny to determine the evolutionary history of duplication in this gene in insects. Next, to ask whether this gene displays behavior consistent with sub/neofunctionalization, we measured gene expression of *Mvl1* and *Mvl2* across eight tissue types in *N. vespilloides*. Finally, we examined expression of the two *Malvolio* copies in head tissue collected from beetles before, during, and after parenting, and found changes in expression during parenting in opposite directions for the two copies. We further compared this expression to RNA-seq data available for *Drosophila melanogaster*. We suggest that the *Malvolio* duplicates in *N. vespilloides* are in the process of evolutionary divergence, with neofunctionalization as a possible endpoint.

## Materials and Methods

### Identification and comparison of protein and gene sequences

To verify the putative duplication that we found in the genome, we searched for putative *N. vespilloides Malvolio* homologs using BLASTp [v2.3.0+; default search settings; Camacho *et al.* (2009)] with *D. melanogaster* (NP_524425.2) and *Tribolium castaneum* (XP_967521.1) *Mvl* sequences. We obtained sequences from the National Center for Biotechnology Information (NCBI) or UniProt databases. We used BLAST on these *Mvl* sequences against the proteome produced from the annotated *N. vespilloides* genome (Cunningham *et al.* 2015b). Further identification of putative *Mvl1* and *Mvl2* of *N. vespilloides* was done using BLASTp with default settings into NCBI’s nonredundant insect protein database, and by BLASTing (BLASTp, default settings) them into *D. melanogaster* and *T. castaneum* proteomes alone to establish if all of sequences were reciprocal best BLAST (RBB) hits for each other.

To visualize protein conservation across both *Mvl* copies, we aligned protein sequences from *N. vespilloides*, *T. castaneum*, *D. melanogaster*, *Homo sapiens*, and *Mus musculus* using ClustalW and produced box shade plots with the Mobyle@Pasteur web portal (http:/mobile.pasteur.fr). We used NCBI protein BLAST with default settings to determine the percent similarity between *N. vespilloides* Mvl1 and Mvl2, and *D. melanogaster* Mvl. The NCBI BLAST used both the sequences for Mvl1 (XP_967521.1) and Mvl2 (XP_973779.1) from *T. castaneum*. Protein sequences were then aligned using Clustal Omega (McWilliam *et al.* 2013), and a model test was performed in MrBayes v3.2 (Ronquist *et al.* 2012) to determine the most appropriate model of protein evolution, which was WAG (Whelan and Goldman 2001).

For the phylogenetic analysis, we included all insect *Mvl* sequences and Mvl proteins we could identify from NCBI, with the exception that we did not include every *Drosophila* spp. Due to the large number of published Drosophila genomes, and to avoid redundancy, we only included *D. melanogaster*. To provide a representative sample of insect species we searched for *Mvl* in Lepidoptera, but there are currently no assembled and annotated genomes of the order that contain a copy of *Mvl*. Thus, our analysis includes all available insect orders. A Bayesian phylogenetic analysis was conducted in MrBayes for 5,000,000 generations with a sample frequency of every 100 generations. The consensus tree was compiled after discarding the first 25% of trees sampled, and the resultant tree was rooted with human and mouse Nramp1 and Nramp2, and a *Crassostrea gigas* Mvl outgroup. We collected all unique Mvl isoform sequences from NCBI for *N. vespilloides*, *T. castaneum*, and *D. melanogaster* and used NCBI protein BLAST at default settings to determine variation between isoform amino acid sequences.

### Comparison of gene expression

We maintained *N. vespilloides* as an actively outbred colony at the University of Georgia. We founded the colony with beetles collected from the wild near the University of Exeter, Cornwall, UK, and new wild individuals were introduced to the colony yearly to maintain genetic variation. We isolated individuals as larvae and housed them individually in 4 × 7 cm biodegradable circular deli containers (Eco products, Boulder, CO) filled with 2.5 cm of moist soil (FoxFarm, Samoa, CA). Individuals were kept in an incubator (Percival Scientific, Perry, IA) set at 22 ± 0.1°, under a 15:9 light:dark cycle. Upon reaching adulthood they were fed two decapitated mealworms (*Tenebrio*) once a week.

For the comparison of expression across different tissues, we collected eight tissues—brain, fat bodies, hindgut, midgut, thoracic musculature, Malpighian tubules, testes, and ovaries—from five virgin female beetles at 26–30 d post adult eclosion (testes came from five males of the same age and rearing conditions). These same tissues types were previously examined for octopamine expression in Cunningham *et al.* (2015a), except for testes, but on separate tissue collections. We dissected beetles in ice cold PBS, starting with the brain and then moving on to the internal organs. We cleaned fat and connective tissue from each organ and placed them in separate 1.5 ml vials with 300 µl of RNAlater (Applied Biosystems, Foster City, CA) on ice. Dissection times for brains were 10 min or less, and the total time of dissections was <30 min. After dissection, we stored organs overnight at 4° and then moved them to −20° until RNA extraction. See Cunningham *et al.* (2015a) for further details.

We collected whole heads from 10 individuals in each of five behavioral states to examine changes associated with changes in behavior: virgins, individuals mated but not provided with the resources necessary to breed, mated individuals provided with a mouse carcass to prepare and that stimulates egg laying, individuals actively caring for and provisioning food to begging offspring, and individuals that had completed parental care and had dispersed away from the carcass and larvae. See Roy-Zokan *et al.* (2015) and Cunningham *et al.* (2016) for further details.

RNA was extracted using a Qiagen RNeasy micro kit (Qiagen, Venlo, The Netherlands) for the brain tissue and larval hemolymph and a Qiagen RNeasy lipid kit for all other tissue. The extractions were performed with 350 µl QIAzol (Qiagen) as the lysis buffer and 150 µl chloroform (J.T. Baker, Center Valley, PA). DNA was removed using DNase I (Qiagen) according to manufacturer’s instructions. After the final RNA product was obtained, it was quantified with the Qubit 2.0 fluorometer (Qubit Systems, Kingston, ON, Canada) according to manufacturer’s instructions. The RNA was then stored until the time of cDNA production in a freezer set to −80°. cDNA was created using 500 ng total RNA and the Quanta Biosciences qScript reverse transcriptase master mix (QuantaBio, Beverly, MA) following the manufacturer’s instructions. The RNA template was then eliminated using RNase H (New England BioLabs, Ipswich, MA) and the single-stranded cDNA was quantified using the Qubit 2.0 fluorometer according to manufacturer’s instructions. The resulting cDNA was then stored at −20°.

Using the two *Malvolio* gene sequences, eight primer pairs (four primer pairs per gene) were produced by utilizing Integrated DNA Technology (IDT, Coralville, IA) and Primer 3 v.4.0 (Koressaar and Remm 2007; Untergasser *et al.* 2012). These primer pairs were then validated by estimating PCR efficiency and observing the number of amplicons generated by each pair. The primer efficiency was determined by running a qRT-PCR reaction with stock cDNA (produced using same methods as experimental cDNA from whole-body samples) diluted to 1:4, 1:16, 1:64, 1:256, and 1:1024 concentrations, while amplicons were observed in the Melt Curve Analysis. These primer pairs had efficiency levels of 1.805 (*Mvl1*) and 1.7852 (*Mvl2*).

The quantification of gene expression was accomplished using a qRT-PCR reaction with the Roche LightCycler 480 using Roche LightCycler 480 SYBR I Green Master Mix (Roche Applied Science, Indianapolis, IN). Each biological replicate (*N* = 5) was run with three technical replicates, using 10 µl reactions containing 5 µl SYBR mix, 2 µl of 1.5 ng/µl cDNA, and 3 µl of an equal mixture of forward and reverse primers at 1.33 µmol/liter each. The LightCycler was run according to manufacturer’s instructions for the enzyme activation step, followed by 45 cycles of amplification at 60° and a disassociation curve step to measure the number of amplicons produced in the reaction. Each reaction included three primers: *Mvl1*, *Mvl2*, and TATA-binding protein as the reference gene (*Mvl1*- forward: CGACGATGACGGGAACTTATG reverse: TTGCGATGGATCTGGTGAAG *Mvl2*- forward: GGTATCGTGGGAGCAGTTATC reverse: GCTGCTCTCGATGAGGTAATAG *tbp*- forward: CACCCATGACTCCAGCAGAT reverse: ACGTGCATGCAGAGCTATCTT).

### Statistical analysis of gene expression

We analyzed the log of the relative expression differences in *Mvl1* and *Mvl2*, where relative expression was quantified as 2^−∆∆^*^C^*^T^ and was relative to expression in ovaries, where expression was negligible for both genes. We made comparisons among the tissues types in JMP Pro 13 (SAS Institute, Cary, NC) using ANOVA on log-transformed relative expression, which was more normally distributed than relative expression, with specific pairwise comparisons made using Fisher’s least significant difference (LSD) test.

Comparisons of expression of *Mvl1* and *Mvl2* across different behavioral states were made as described in Roy-Zokan *et al.* (2015) and Cunningham *et al.* (2016), using 2^−∆∆^*^C^*^T^ with relative expression standardized to virgins. We used virgins as the comparison as this is the behavioral/physiological state of individuals used in the tissue comparison, and that we have used in previous studies as a nonsocial state. ANOVA on log-transformed relative expression was used to determine statistically significant changes in expression.

To provide comparative context for our results on expression in different behavioral states and tissues, we collected *D. melanogaster* RNA-seq tissue data from FlyBase (FBrf0221009) (Gelbart and Emmert 2013). We then averaged normalized expression across each exon to produce one value for each tissue and behavioral state. These data were then visualized in JMP Pro 13, allowing descriptive comparisons with our expression patterns in *N. vespilloides*.

### Data availability

All data and reagents are available on request. Data were deposited in Dryad (doi: 10.5061/dryad.110qd). All accession numbers for sequences used in the phylogenetic comparison are available in Supplemental Material, File S1.

## Results

### Phylogenetic analysis of Malvolio across insects

**Figure 1:**
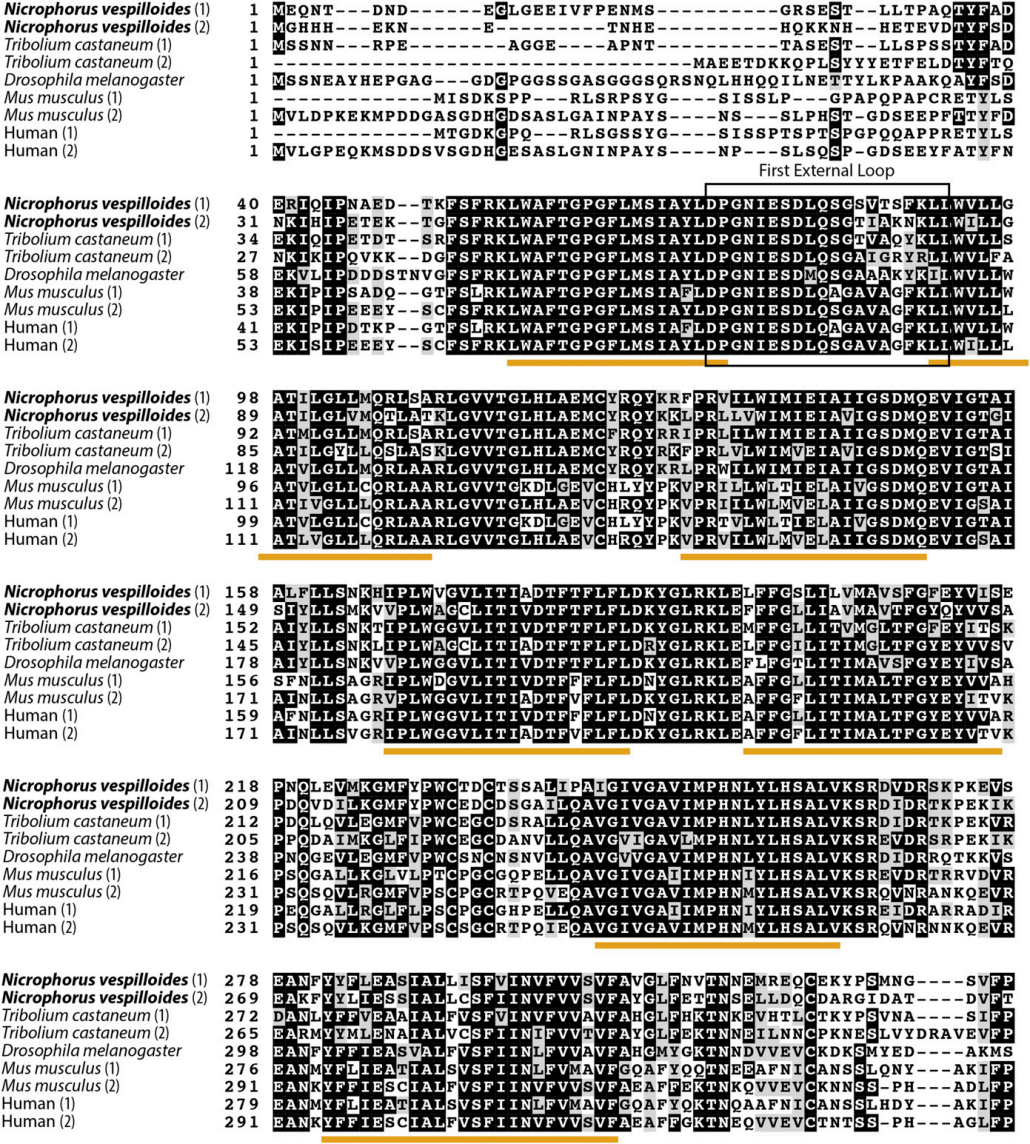

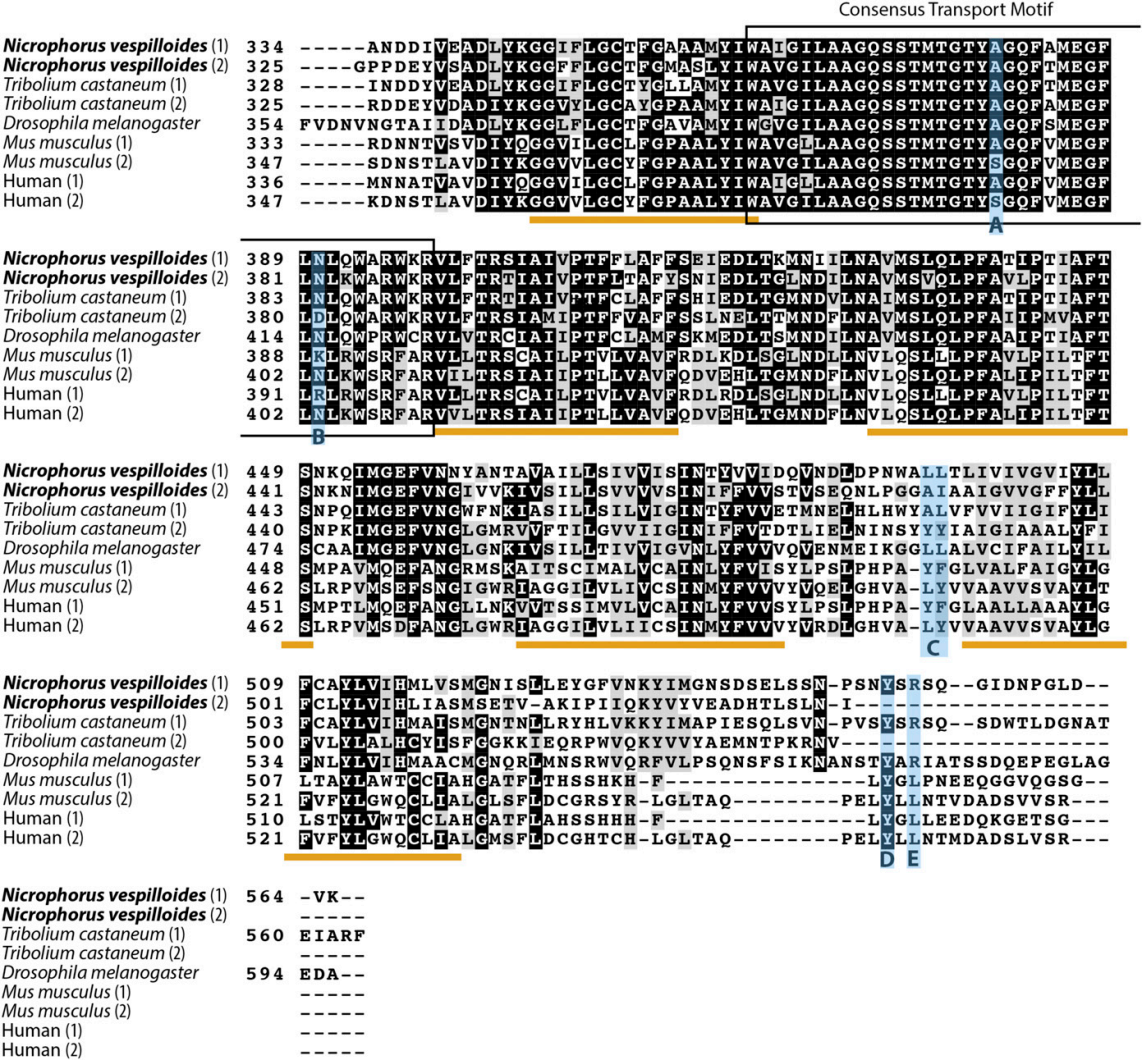
Amino acid alignment of *N. vespilloides* Mvl1 and Mvl2, *T. castaneum* Mvl1 and Mvl2, *D. melanogaster* Mvl, and the homologous *M. musculus* and human Nramp1 and Nramp2. Shaded regions represent a >50% similarity among sequences. Underlined sequences indicate putative transmembrane domains, while other regions of interest are indicated by labeled boxes. Specific amino acids of interest are highlighted and labeled with a letter. A, B, and C represent amino acids that indicate symporter or antiporter activity, with the following patterns; A:S indicates symporter, A indicates antiporter, B:N or D indicates symporter, K or R indicates antiporter, C: LY indicates symporter, and YF indicates antiporter. D and E are conserved amino acid positions that are part of the intracellular localization motif, which determines the intracellular localization of the protein in humans (Tabuchi *et al.* 2002).

Box shade plots illustrating sequence homology between *N. vespilloides*, *T. castaneum*, *D. melanogaster*, *H. sapiens*, and *M. musculus* indicate a high level of conservation between the different species, especially in the transmembrane regions (Figure 1). The first external loop and the consensus transport motif are highly conserved as well. The primary differences between the proteins are observed in the N-terminal and C-terminal ends. Overall, *N. vespilloides* Mvl1 shared 66% similarity and Mvl2 shared 64% similarity with *D. melanogaster* Mvl.

*N. vespilloides* Mvl1 has two isoforms differing in only the last several amino acids at the C-terminal end. The intracellular localization motif, which in humans is composed of the amino acids located at positions 555 (D on Figure 1) and 557 (E on Figure 1) and has been implicated in the intracellular localization of Nramp2, marks the beginning of the alternative splice site. The amino acid at the insect equivalent of human position 555 is always a Y; meanwhile, the amino acid at the equivalent of position 557 differs between the two isoforms, with isoform X1 having an R and isoform X2 having an S. This pattern is also seen in the isoforms of *T. castaneum* Mvl1 and *D. melanogaster* Mvl, with each isoform having a Y at the 555 position, and either an R or an S at the 557 position. Neither *N. vespilloides* nor *T. castaneum* Mvl2 has different isoforms.

**Figure 2:**
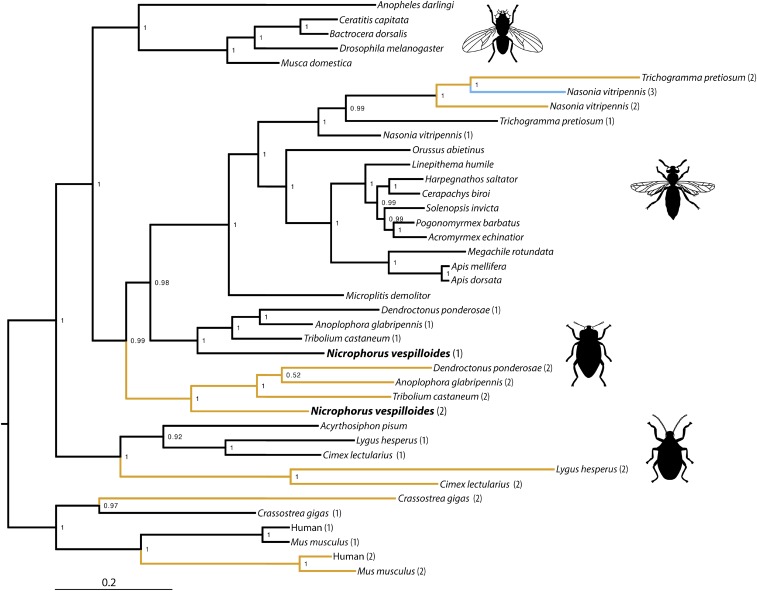
Phylogenetic relationships of *Malvolio*. Duplications are indicated with yellow and major insect groups are indicated pictorially. Included in this tree are mammals [human (*Homo sapiens*) and mouse (*Mus musculus*)] and oyster (*Crassostera gigas*) as outgroups, and all insect orders where we could find homologs of *Malvolio* including Hemiptera [bed bug (*Cimex lectularius*), pea aphid (*Acyrthosiphon pisum*), and western tarnished plant bug (*Lygus hesperus*)], Hymenoptera (bees: *Apis dorsata*, *Apis mellifera*, *Metamicroptera rotundata*; wasps: *Orussus abietinus*, *Microplitis demolitor*, *Nasonia vitripennis*, *Trichogramma pretiosum*; ants: *Acromyrmex echinatior*, *Pogonomyrmex barbatus*, *Solenopsis invicta*, *Ooceraea biroi*, *Harpegnathos saltator*, *Linepithema humile*), Coleoptera (beetles: *Tribolium castaneum*, *Nicrophorus vespilloides*, *Anoplophora glabripennis*, *Dendroctonus ponderosae*), and Diptera (flies: *Ceratitis capitata*, *Bactrocera dorsalis*, *Drosophila melanogaster*, *Musca domestica*, *Anopheles darlingi*).

Our phylogenetic analysis (Figure 2) shows that *Mvl* has undergone several independent gene duplications that have been maintained both in insects and other animals. Among insects, *Mvl* appears to have duplicated separately in hemipterans (true bugs), Coleoptera (beetles), and wasps. Other than wasps, among the Hymenoptera, bees and ants have only one copy of *Mvl*. The coleopteran duplication appears to have preceded the split of beetles and Hymenoptera, which have lost *Mvl2*. Therefore, *Mvl1* shows greater homology to all hymenopteran *Mvl* genes.

### Tissue-specific expression

**Figure 3:**
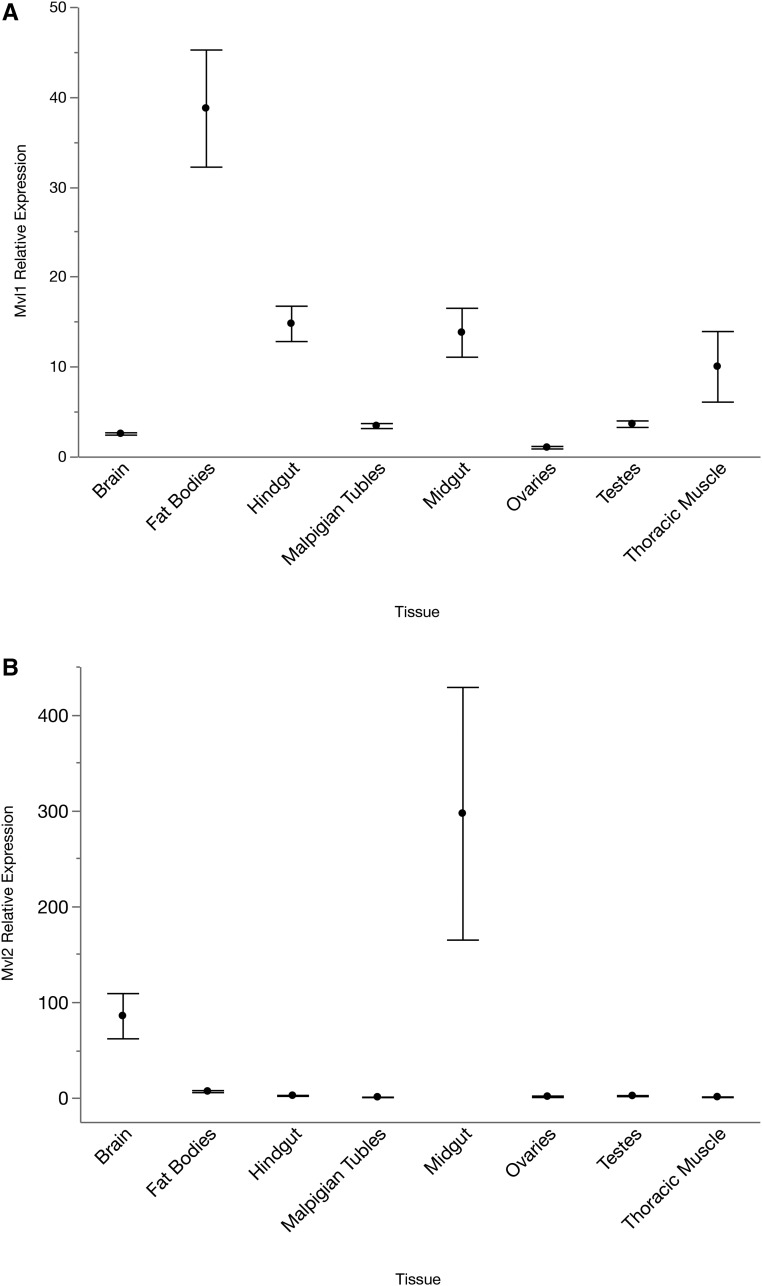
Relative expression of *Mvl1* (A) and *Mvl2* (B) in different tissue types. Two extreme values of *Mvl2* relative expression in the midgut were removed; removal of these two does not alter any statistical interpretations. Means ± 1 SE are presented.

Expression of *Mvl1* in brain, fat bodies, Malpighian tubules, midgut, ovaries, testes, and thoracic musculature varied across the different tissue types (*F*_7,32_ = 44.361, *P* < 0.0001). Expression in fat bodies was statistically significantly higher than in other tissues (Figure 3a). Hindgut, midgut, and thoracic musculature had moderate levels of expression, while expression was relatively low in testes, Malpighian tubules, brains, and ovaries.

Expression patterns across tissues of *Mvl2* differed from those of *Mvl1* (Figure 3b). Overall, there was statistically significantly different expression across the different tissue types (*F*_7,32_ = 37.420, *P* < 0.0001) although in all tissues expression was much lower than that of *Mvl1*. Expression was highest in midgut and brain, with low expression in fat bodies, hindgut, and testes. Expression was negligible, and sometimes undetectable, in Malpighian tubules, ovaries, and thoracic muscle (Figure 3b).

### Expression across different behavioral states

**Figure 4:**
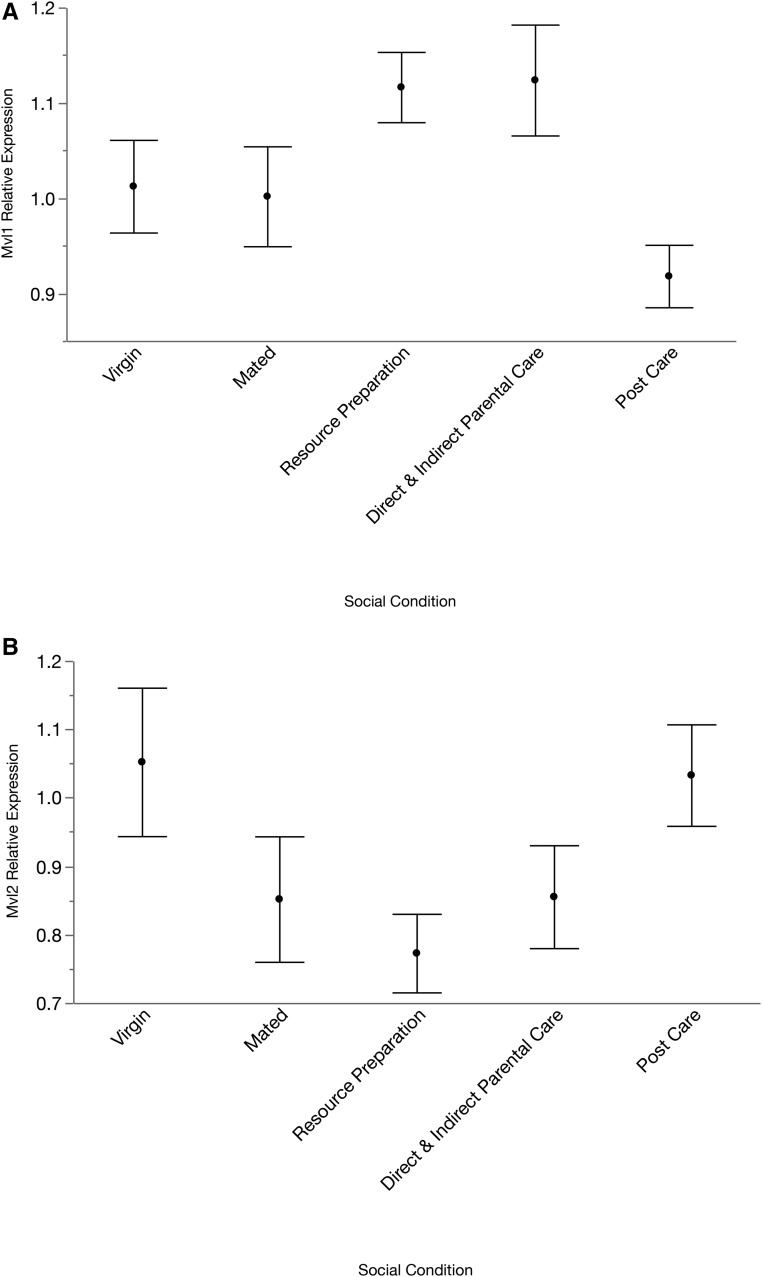
Relative expression of *Mvl1* (A) and *Mvl2* (B) in female heads across different physiological/behavioral states. Virgin females had no social experience or exposure to a carcass resource required for mating and oogenesis; mated females were placed with a male for 48 hr but not provided with a carcass; resource preparation were mated females provided with a carcass for 48 hr to prepare for reproduction and provisioning of offspring; direct and indirect care were females sampled during the most active period of direct feeding of offspring and in the act of regurgitating food to the offspring; post care females had dispersed from the carcass and had no further interactions with larvae for at least 24 hr. All individuals were 21 d of age when sampled. Means ± 1 SE are presented.

Expression patterns across behavioral states differed for *Mvl1* compared with *Mvl2*, with the patterns of expression being opposite for *Mvl1* and *Mvl2* across these states. Overall, there were statistically significant changes in expression across the behavioral states in *Mvl1* (*F*_4,45_ = 3.4087, *P* = 0.0162) (Figure 4a), with a significant increase in expression in resource preparation (*P* = 0.0044) and caring for offspring (*P* = 0.0032). There was no overall statistically significant difference in expression across behavioral states for *Mvl2* (*F*_4,43_ = 2.2682, *P* = 0.077) (Figure 4b) although expression decreased during social interactions, and resource preparation showed significantly lower expression than either virgin (*P* = 0.019) or post care (*P* = 0.0285).

RNA-seq data from *D. melanogaster* shows a pattern of relatively stable *Mvl* expression over time in the heads of virgin females, while the heads of mated females have a steady decrease in *Mvl* expression. Thus, the single copy of Drosophila *Mvl* has a similar expression to *N. vespilloides Mvl2* in the brain during mating. By contrast, Drosophila *Mvl* expression increases several-fold in the ovaries of mated females compared with virgin females. Drosophila *Mvl* expression in the digestive system and in testes is similar to *Mvl1* expression in *N. vespilloides* (Figure S1).

## Discussion

Gene duplication is a major factor in evolution (Ohno 1970; Innan and Kondrashov 2010; Wagner 2011), particularly where there is neofunctionalization, as the duplicated gene can permit access to variation that may have otherwise been constrained. Here we examined *Malvolio*, a gene that typically functions as a transporter of divalent cations. Examining the genome of the subsocial beetle, *N. vespilloides*, we found that *Malvolio* was duplicated in this insect. Two other factors suggested that it would be informative to examine this duplication further: first, *Malvolio* is the homolog of *Nramp* in vertebrates, a gene that is duplicated and subfunctionalized (Techau *et al.* 2007; Neves *et al.* 2011) and second, *Malvolio* is known to play a role in social behavior in bees (although they have only one copy), suggesting that a duplication could therefore be sub/neofunctionalized for behavior. Our study had two components, the first analyzing and comparing Mvl protein sequence and the second examining expression of the duplicated genes. These studies provide several lines of evidence supporting the neofunctionalization of Mvl in *N. vespilloides* specifically, suggesting that Mvl1 has retained a more ancestral function while Mvl2 has diverged.

Our phylogenetic analysis showed that many species of insects have duplicate copies and, furthermore, that these duplications appear to have occurred in multiple lineage-specific events. *Malvolio* duplicates have arisen and persisted in at least three different insect lineages (and in wasps, a second duplication event appears to have led to at least one species having three copies of *Malvolio*). Given the tendency of duplicated genes to remain redundant and eventually be removed from the genome, this suggests that *Malvolio* may possess qualities that have been found to encourage persistence after a duplication event (Kondrashov *et al.* 2002; Papp *et al.* 2003; Davis and Petrov 2004; Jordan *et al.* 2004; Marland *et al.* 2004).

The first evidence supporting sub/neofunctionalization of the *Mvl* genes specifically in *N. vespilloides* comes from analysis of the comparative structure of both copies. Specifically, we examined four regions of the gene: the first external loop, which is responsible for metal ion binding specificity (Cohen *et al.* 2003; Southon *et al.* 2008); the consensus transport motif, which is important for the formation of voltage-gated potassium channels (Cellier *et al.* 2007); a region indicating whether the protein will function as a symporter or transporter (Techau *et al.* 2007; Southon *et al.* 2008); and the C-terminal region, which determines intracellular localization (Tabuchi *et al.* 2002). The first three of these regions are highly similar between Mvl1 and Mvl2 in *N. vespilloides*, suggesting that there may be overlap in transport specificity and mechanism. However, there does appear to be significant variation in the C-terminal region. Specifically, our alignment of Mvl and Nramp proteins suggests that amino acids known to affect localization of human Nramp2 (Tabuchi *et al.* 2002) are conserved in *N. vespilloides* Mvl1 but completely absent in Mvl2. Furthermore, Mvl1 undergoes alternative splicing at this site, which suggests that, as in humans (Tabuchi *et al.* 2002), different splice forms of Mvl1 may locate to different parts of the cell. These localizations are likely different from those of Mvl2, where these amino acids are absent. This predicted variation in cellular localization would support a divergence in tissue- or organ-level function between Mvl1 and Mvl2 despite the overall similarity between these gene copies.

We next examined tissue-specific expression of both genes to further investigate whether sub/neofunctionalization may be responsible for the maintenance of both *Malvolio* duplicates in *N. vespilloides*. Our data show that *Mvl1* is expressed in all eight measured tissues, with relatively low variance in gene expression within a tissue. In contrast to *Mvl1*, expression of *Mvl2* was limited to only two tissues, the brain and the midgut. This pattern is roughly consistent with tissue- and stage-specific data from *Tribolium*, which also shows high and ubiquitous *Mvl1* expression as opposed to low and inconsistent, but detectable, *Mvl2* expression (Dippel *et al.* 2014). This suggests that *Mvl1* may have maintained a conserved homeostatic role throughout the coleopteran lineage, consistent with the necessity of manganese transport on the cellular level (Culotta *et al.* 2005). Differences in expression between specific tissues may be related to other well-established functions of *Mvl* and its homologs, such as intracellular immunity (Evans *et al.* 2001; Cellier *et al.* 2007). *Mvl2*, on the other hand, appears not to be required for basic tissue function, and thus may be subject to weaker pleiotropic constraints.

Finally, we examined the expression patterns of both genes in the head, which includes both brain and fat body, in relation to reproductive and parental care behavior to further examine the possibility that the function of *Mvl2* has diverged from that of *Mvl1* in *N. vespilloides*. This species of beetle is not a genetic model organism, although we have a sequenced genome (Cunningham *et al.* 2015b). Instead, *N. vespilloides* is biologically interesting for its unusually elaborate parenting and social interactions (Parker *et al.* 2015). Previous research has shown that genes differentially expressed during parenting are detected in the specific social conditions we sampled (Parker *et al.* 2015; Roy-Zokan *et al.* 2015; Cunningham *et al.* 2016, 2017). Our hypothesis that there may be a behavioral function was based on the studies showing *Malvolio* is involved in caste differentiation in honey bees (Ben-Shahar *et al.* 2004) as well as feeding behavior in *Drosophila* (Søvik *et al.* 2017). We have hypothesized that feeding pathways are coopted to influence parental provisioning behavior (Cunningham *et al.* 2016, 2017), which fits the known roles of *Malvolio*, making this gene a strong candidate for influencing parenting. We found that the two copies do show differences in expression in head tissue associated with changes in behavior and social interactions. Whereas *Mvl1* expression increases during parenting, *Mvl2* appears to decrease during the same behavioral stages. These opposing expression patterns suggest that even though both gene copies have retained roles in social behavior, these roles have diverged.

Drosophila *Mvl* expression in the head shows a similar trend to *Mvl2* in *N. vespilloides*, where mated individuals have decreased expression. Interestingly, this is the opposite of *Mvl1* in *N. vespilloides*, where the expression increased after mating. The relatively high expression of *Mvl*2 in the midgut of *N. vespilloides* likely indicates a role in digestive iron intake, though further experiments would be required to determine if this function is independent or complementary to *Mvl*1 in the midgut.

Given the tissue- and stage-specific expression patterns of *Mvl1* and *Mvl2*, it appears likely that these genes have undergone neofunctionalization in burying beetles. Definitive evidence will require functional studies. It is intriguing that expression is opposite for *Mvl*1 and *Mvl*2 during parent-offspring social interactions. Data from honey bees, in which *Malvolio* is not duplicated, show that a single copy can account for both behavioral and other gene functions (Ben-Shahar *et al.* 2004; Søvik *et al.* 2015), suggesting that divergence in gene function between copies could be obtained by subfunctionalization alone. However, if this were the case, we would predict that one copy would have completely lost its association with behavior in *N. vespilloides*. Instead, we observe the evolution of opposing gene expression patterns between copies, meaning the expression patterns of at least one gene copy must be derived. Given the highly divergent and elaborate social interactions during parenting, and extensive parenting, in this species, this suggests that *Malvolio* may be coopted for further behavioral evolution. Furthermore, divergence in tissue-specific expression patterns, as we observed here, is often associated with neofunctionalization (Huminiecki and Wolfe 2004, Li *et al.* 2005). Therefore, our data are consistent with neofunctionalization. It may be that the expression patterns of *Mvl2* in the brain and midgut are still evolving, and understanding whether expression is being gained or lost in these tissues along with explicitly functional studies would help resolve this question.

In conclusion, *N. vespilloides* produces two copies of *Malvolio*, a gene which is commonly duplicated and maintained in insects and vertebrates. Our sequence and expression data suggest that, in *N. vespilloides*, *Malvolio* has experienced neofunctionalization following its duplication, potentially with an enhanced role in behavior. Although further functional studies are needed to eliminate subfunctionalization as an explanation for the patterns we see, our data do suggest that the two copies are not equivalent. Finally, we suggest that the predilection for duplicates of this gene to be maintained may reflect a tendency for sub- or neofunctionalization of *Mvl* in other systems as well.

## Supplementary Material

Supplemental material is available online at www.g3journal.org/lookup/suppl/doi:10.1534/g3.117.300183/-/DC1.
