# Supplementary material for: Duplication and Sub/Neofunctionalization of Malvolio, an Insect Homolog of Nramp, in the Subsocial Beetle Nicrophorus vespilloides
Source: G3 (Bethesda). 2017 Aug 22;7(10):3393–403. doi: 10.1534/g3.117.300183 (PMC5633388; doi:10.1534/g3.117.300183)

Supplementary Figure S1: Expression of Mvl in different tissues, stages, in *Drosophila melanogaster*. Data from Flybase (Gelbart and Emmert, 2013. http://flybase.org/reports/FBrf0212041.html). Data analyzed here are deposited in Dryad.

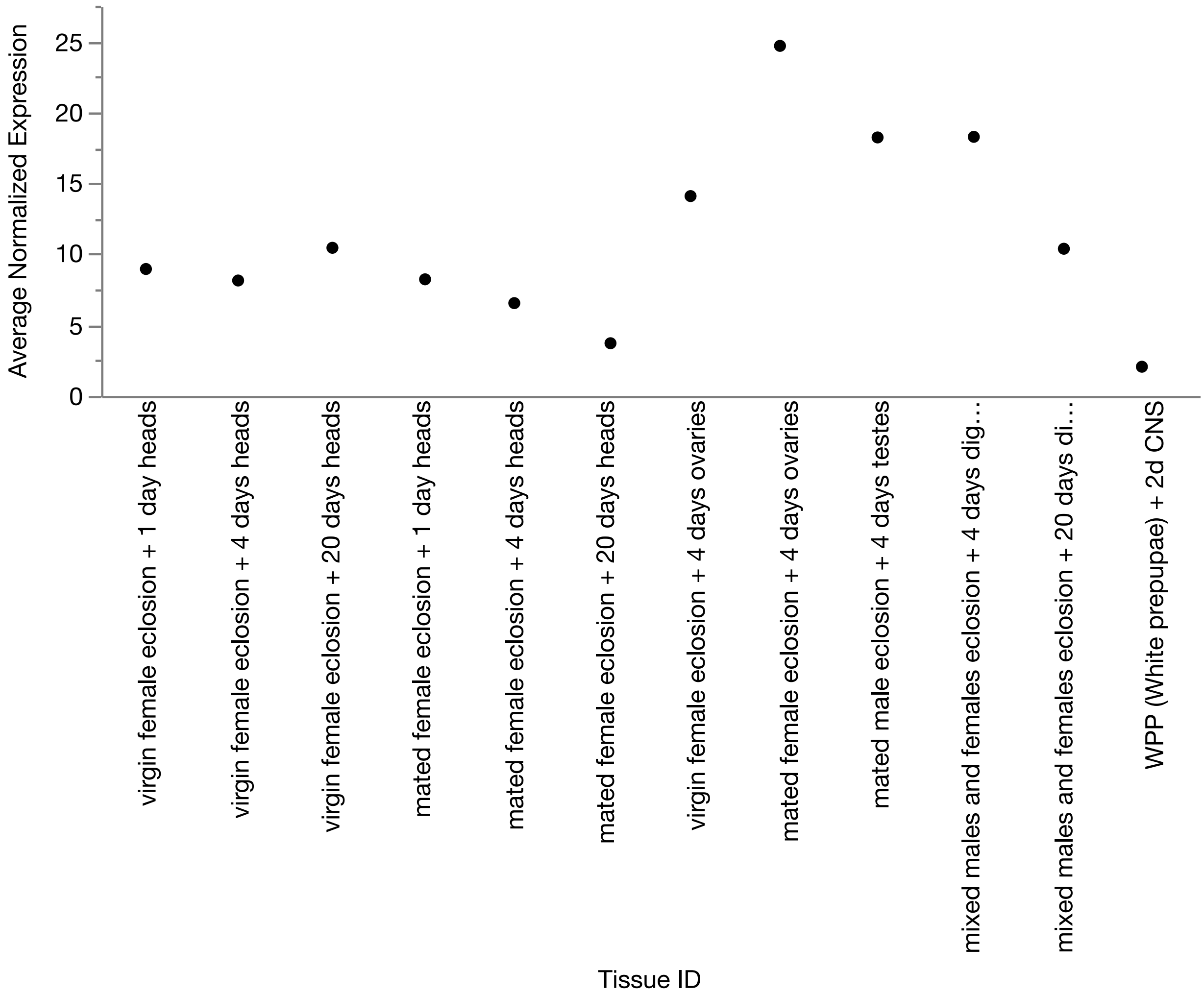

Supplement: Supplementary file 1 [file 3393FigureS1.pdf]
